# Advances in European Psychotraumatology

**DOI:** 10.3402/ejpt.v3i0.20249

**Published:** 2012-12-21

**Authors:** Miranda Olff

*European Journal of Psychotraumatology* (*EJPT*) is now completing its third volume. It is exciting to see how the range of articles addressing both clinical issues as well as more basic research, include samples of simple and complex PTSD as well as other disorders such as substance use disorder (e.g. Gielen et al., 2012) or posttraumatic panic attacks (Joscelyne et al., 2012). The populations studied range from terrorist attack (Thoresen et al. 2012), disaster victims (Grimm et al., 2012) and refugees (e.g. Zepinic et al., 2012), to civilian war victims (e.g. Palgi et al.,) and veterans (Kitchiner et al., 2012), but also include journalists (e.g. Backholm & Björkqvist, 2012) and legal representatives of asylum seekers (Wilson-Shaw et al., 2012). The life span is covered with study populations ranging from children (e.g. Wittman et al., 2012) to the elderly (Glück, et al., 2012). Designs differ from large epidemiological studies (e.g. Karanci et al., 2012) to smaller innovative pilot studies (e.g. Poundja et al., 2012) or experimental setups (e.g. Sack et al., 2012). Biological measures are included, e.g. De Kloet et al. (2012) examine neuroendocrine and immunological measures in PTSD, as well as validation studies of questionnaires in important European languages such as French (de Fouchier et al., 2012) and Polish (Dragan et al., 2012). Well established trauma-focused interventions like exposure therapy are scrutinized for their use in clinical practice, e.g. Palgi et al., 2012, and newer interventions e.g. adding biofeedback (Morina et al., 2012) are explored for effectivity, in sum reflecting the wide variety of European and in fact worldwide psychotraumatology research.

***Highlights of 2012 –*** Among the many interesting papers published during 2012 I would especially – and for different reasons – like to mention the following four:

1. Systematic review and meta-analyses of psychosocial interventions for veterans of the military by *Neil J. Kitchiner, Neil P. Roberts, David Wilcox, Jonathan I. Bisson* (Kitchiner et al., 2012).

While consulting the editorial board this paper came out as the top paper of 2012. Although clearly one of my favourites, being a scientist I should mention that limitations of my ‘poll’ were the brief time period I had given to respond and possibly a recency effect as this paper was published just a few days before writing this editorial. That said, a systematic review/meta-analysis by definition provides an overarching synthesis of findings that individual papers can never provide and therefore moves the field forward. This comprehensive review of treatment methods for mental health problems following exposure to combat in veterans with often complex psychiatric problems is timely as the treatment evidence for this group is still inadequate (Institute of Medicine Committee, 2007). The authors show that although there was significant clinical and statistical heterogeneity in the included studies and results should thus be interpreted cautiously, the meta-analyses limited to the clinically more homogeneous trauma-focused interventions were positive. Veterans respond to out-patient trauma-focused psychosocial interventions for chronic PTSD on a one-to-one or group basis (with the therapist within the same room), which is consistent with the evidence from meta-analyses of civilian studies (Bisson et al., 2007) and supports a recommendation that trauma-focused interventions should be offered to all veterans with chronic PTSD.

2. Examining potential contraindications for prolonged exposure therapy for PTSD by *Agnes van Minnen, Melanie S. Harned, Lori Zoellner, Katherine Mills* (Van Minnen et al., 2012).

We are all aware of the fact that PTSD has high rates of comorbidity. In fact more often than not we see PTSD comorbid with other disorders. Nonetheless, little is known about whether evidence based treatments like prolonged exposure are indicated for these patient groups. The authors of this paper, international exposure therapy experts from different continents, conclude that prolonged exposure can be safely and effectively used with patients with comorbidities such as major depression, dissociation, borderline personality disorder, psychosis, suicidal behaviour and non-suicidal self-injury, or substance use disorders. However, they do recommend that in cases with severe comorbidity to treat PTSD while providing integrated or concurrent treatment to monitor and address the comorbid problems.

3. PTSD and trauma in Austria's elderly: influence of wartime experiences, postwar zone of occupation, and life time traumatization on today's mental health status - an interdisciplinary approach by *Tobias M. Glück, Ulrich S. Tran, Brigitte Lueger-Schuster* (Gluck et al., 2012).

In this article the current ESTSS president Brigitte Lueger-Schuster and her team have addressed the long term traumatization due to World War II experiences in elderly persons in Austria showing that PTSD is a common disorder in the elderly due to these experiences. They also point to the importance of post war distressing conditions in posing a further risk for symptomatology and distress in later years. The authors recognize that with a growing proportion of older people in Europe's societies, PTSD cannot be ignored and sequelae of trauma need to be considered in treatment and care.

4. Acute dissociation and cardiac reactivity to script-driven imagery in trauma-related disorders by *Martin Sack, Melanie Cillien, James W. Hopper (*Sack et al., 2012).

The immediate past president of the DeGPT, the traumatic stress society representing the German language area, Martin Sack, and his team examined potential acute protective functions of dissociation using an experimental design. They assessed psychophysiological reactions during exposure to script-driven trauma imagery in relation to acute responses of reexperiencing and dissociative symptoms and found that acute dissociative reactions are a potential moderator of the stress response to experimental paradigms. Here also the age during traumatization appeared to be important with early life trauma leading to an increased risk for suffering from dissociative symptoms or dissociative disorders during later life.

Other papers worth special mention include:

Assessment of anhedonia in psychological trauma: psychometric and neuroimaging perspectives by *Paul A. Frewen, David J.A. Dozois, Ruth A. Lanius* (Frewen et al., 2012), which investigated negative affective responses to what would normally be considered pleasant events (e.g., receiving a compliment or gift, physical affection) in traumatized persons using the Hedonic Deficit & Interference Scale and a neuroimaging paradigm. The findings may be relevant to studies examining the effectiveness of treatments in the reduction of hedonic deficits and negative affective interference.

Refugees’ views of the effectiveness of support provided by their host countries by *Vito Zepinic, Maria Bogic, Stefan Priebe* (Zepinic et al., 2012), providing insight into the social context of life after trauma. The breadth of this paper may be appealing to anybody who works in trauma research.

The overlooked relationship between motivational abilities and posttraumatic stress: a review by *Keti Simmen-Janevska, Veronika Brandstätter, Andreas Maercker* (Simmen-Janevska et al., 2012) showing that, despite a lack of research investigating motivational factors as outcome variables following traumatic experiences, motivational constructs such as self-efficacy, locus of control, self-esteem, impulsivity/self-control, seem to predict posttraumatic stress over the life span.

***Thematic clusters –*** In this volume we introduced the *“Thematic cluster”*, the Open Access variant of a Special Issue (Olff, 2012). The first thematic cluster was edited by *Vittoria Ardino* on the role of trauma and PTSD in offending behaviour, addressing specific trajectories that connect trauma and PTSD to criminal behaviour (Ardino, 2012), female juvenile offenders’ trauma-related mental health and rehabilitation issues (Foy et al., 2012) as well as the challenges and promises of trauma-informed correctional care and how this translates into strategies for administrative support, staff development, programming, and relevant clinical approaches (Miller & Najavits, 2012).

***Conference abstracts–*** The International Society of Psychoneuroendocrinology with *Rachel Yehuda* as President and *Tom Hildebrandt* as Program Chair organized their 42nd Annual Conference on: Effects of Traumatic Stress - Molecular and Hormonal Mechanisms. An exciting meeting with an opening session on the 9/11 attacks with a view on the 9/11 memorial in New York. This resulted in an online abstract book in *EJPT* (Suppl 1, 2012) with each author's contribution indexed with an individual DOI. This was not only helpful to the conference participants but also made the content of this meeting available all over the world. Next year we will be publishing the abstracts and keynote lectures from the ESTSS biennial congress in Bologna June 6-9.

***Inaugural lectures –*** In this volume we have also introduced papers based on *inaugural lectures* (Engelhard, 2012; Olff 2012). These are contributions based on important events such as the honorary lecture of a newly appointed professor, the keynote of a major conference in the area of traumatic stress, or similar.

***Who is reading EJPT –*** Since the journal's launch in December 2012, the high quality content has attracted 42, 000 visits from readers in 150 countries, with most visits coming from the USA, the Netherlands, and the UK, followed by Germany, Sweden, Canada, Australia, Switzerland, Norway and India. We have noted 26,000 unique visitors, and 37%, or 15,500, of the visits have been paid by so-called returning visitors. This means that a considerable number of interested researchers, clinicians and others are returning to the *EJPT* website, primarily – one would assume – to check new content or retrieve older articles. Considering that the journal has only been in existence for two years we think it is safe to say that Open Access does indeed mean wide dissemination of research results – and therefore also of the knowledge we need to better understand the consequences of psychotrauma and to constantly improve the treatment of people who have been traumatized.

***Indexing and impact factor –*** The high quality of the papers published in *EJPT* has relatively quickly led important indexing services to include the journal in their collections. Most importantly, in 2012 the journal was included in PubMed Central, the free digital archive of biomedical and life sciences journal literature at the US National Institutes of Health (NIH), developed and managed by NIH's National Center for Biotechnology Information in the National Library of Medicine. This means that *EJPT* is now also covered by PubMed. We are expecting a verdict from Psychinfo shortly.

Thomson Reuters (ISI) has received an application from us for inclusion in the Web of Science and Science Citation Database, and we hope *EJPT* will receive its first impact factor in 2014 or 2015. We already see that some of the papers we publish are receiving citations. Thus, for instance, the impact of “Taxing working memory on negative and positive memories” by Iris M. Engelhard and co-workers (Engelhard et al., 2010) has already received 18 citations, and “Fear conditioning and early life vulnerabilities: two distinct pathways of emotional dysregulation and brain dysfunction in PTSD” by *Ruth A. Lanius* and her group (Lanius et al., 2010) has so far received 15 citations.

***Hello, buenos días, bonjour, guten Tag**,* 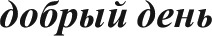 ***buongiorno, dobry dzień, iyi günler –*** As we represent many different languages we very much value our translations of abstracts and sometimes full papers. Do not forget to check this on the website under supplementary material.

***Editorial board –*** A new Associate Editor joined the team in 2012, Marylène Cloitre. She is Past President of the International Society for Traumatic Stress Studies (ISTSS) and founder and director of The Institute for Trauma and Stress at the NYU Child Study Center. An expert in the field of traumatic stress Marylène has published extensively on the treatment of survivors of childhood abuse. She will join forces with me and the other Associate Editors, Vittoria Ardino, Chris Brewin, Ruth Lanius, Agnes van Minnen, Rita Rosner, and Stuart Turner, and we will all—with our different types of expertise and background – continue to secure the highest quality of the papers to be published in the 2013 volume.

***Collaboration with other STSSs –** European Journal of Psychotraumatology* is the official journal of the European Society for Traumatic Stress Studies (ESTSS) and as such embedded in a professional society, which not only guarantees its legitimacy and high standards but also its integrity, being a **non-profit** publication. In addition, we work closely together with the ISTSS and other societies of traumatic stress (STSS) around the world. For instance, we are part of the Global Collaboration on Traumatic Stress project, a joint effort of all STSS's around the world to simultaneously push a single clearly identified topic, this year ‘Child abuse and its latent impact’.

***Open Access – what does it mean?*** Open Access, as defined as both free access to research results as well as the right to re-use the literature and data found as long as the original source is cited, contributes to the creation of a new information infrastructure. This infrastructure offers opportunities for researchers to connect with one another and the broader world. As noted above, the broad dissemination of *EJPT* supports this view.

“Publish open access or perish?” is the title of a forthcoming symposium to be held February 2013 by the KNAW (Royal Netherlands Academy of Arts and Sciences). Increasingly, institutions and funding bodies are recognizing the importance of supporting Open Access. This past summer two highly visible policies were put forth. The European Commission presented legislation for the implementation of the next research programme – Horizon 2020 – which included an Open Access mandate and funding to cover article processing charges in all areas of research supported by the EC. In the United Kingdom, the so-called ‘Finch Report’ was presented, which also included funding to cover article processing charges, and the recommendations in the report were adopted by the Research Councils UK. While both of these policies are currently being debated, they both illustrate a more pronounced support for new forms of disseminating research results.

Thus we enter into the fourth volume of *EJPT* with much confidence and aim to develop the journal even further. Tune in next time at this time of the year to hear more about our progress! Meanwhile you are welcome to submit your contribution to the journal whether you are a member of ESTSS or not.

*Miranda Olff* Editor-in-Chief
